# Sonographic Estimation of Normal Palatine Tonsil Size in Under 18 Year Olds Who Visited a Tertiary Teaching Hospital in Addis Ababa, Ethiopia

**DOI:** 10.1155/rrp/9996789

**Published:** 2025-09-15

**Authors:** Raja Tamiru Muleta, Natnael Alemu Bezabih, Michael Teklehaimanot Abera, Henok Dessalegn Damtew, Fathia Omer Salah, Yocabel Gorfu G/Medihn, Erko Chala Beyene

**Affiliations:** Department of Radiology, College of Health Sciences, Addis Ababa University, Addis Ababa, Ethiopia

**Keywords:** palatine tonsil, palatine tonsil volume, pediatric tonsil size, tonsil size variability, tonsil ultrasonography

## Abstract

**Background:** Currently, there is no standard normal tonsillar measurement using ultrasound (US) which can be used for comparison with an abnormal palatine tonsil in clinical practice.

**Objectives:** In this study, we aim to estimate normal palatine tonsil size using US in children who have no tonsillar-related disease in TASH, Addis Ababa, Ethiopia.

**Methods:** A prospective cross-sectional study was conducted at TASH. The study was conducted on patients under age of 18 years who visit the hospital for complaints unrelated to tonsillar diseases from May 26–September 30, 2023. A structured research tool was used to collect all the necessary data from participants selected by convenience sampling. Data were entered and analyzed using SPSS Version 27 software.

**Results:** A total of 265 patients were enrolled in the study in the age range of 0–18 years. The mean palatine tonsillar volume ± SD for the right palatine tonsil was 0.97cm^3^ ± 0.55 and it was 0.97cm^3^ ± 0.56 for the left tonsil. We did not identify any significant correlation between PT values and sex as well as the side of palatine tonsil measured. We detected a significant positive correlation between PT values and height, weight, and body mass index (BMI), height having the strongest correlation and BMI the least. Relative contribution of anthropometric parameters to tonsil size varies with age: height is more influential in younger children and weight in early childhood, whereas evidence for BMI influence in older individuals is inconclusive.

**Conclusion:** Normal tonsillar sizes on transcervical US can be used to aid diagnosis of tonsillar pathologies. Palatine tonsil size correlates with age, height, weight, and BMI; however, no correlation is present for sex and side. Age-specific anthropometric profiles and ethnic population differences also contribute to variability in palatine tonsil size measurements and should be carefully considered when interpreting normal tonsil dimensions.

## 1. Introduction

The right and left palatine tonsils are immunocompetent solid lymphoid glands in the oropharyngeal isthmus located between the palatoglossal and palatopharyngeal arches [1, 2]. They have irregular surface due to their ingrowing surface epithelium called tonsillar crypts.

There are different methods of assessing the tonsillar size. Among these, physical examination and inspection have been traditionally used. Although palatine tonsils are directly visible during oral examination, ultrasonography offers objective, reproducible, and quantitative measurements of tonsil dimensions that visual inspection alone cannot reliably provide. Ultrasound (US) enables measurement of depth and three-dimensional size, including transverse and longitudinal diameters, which are difficult to assess accurately by sight or palpation. Moreover, US can assess echogenicity changes that help differentiate normal from inflamed or pathological tonsils, providing diagnostic information beyond mere size [3].

In spite of wide employment of magnetic resonance imaging (MRI) and computerized tomography (CT) to assess palatine tonsil pathologies, their use in the pediatric population is limited by ionizing radiation, need for sedation, and patient transport, as well as high cost [4, 5].

Recently, US has been increasingly used to diagnose tonsillar pathologies and evaluate the morphological and volumetric changes of tonsils [6]. The application is particularly progressing in the pediatric population and US measurement of tonsillar volume correlates well with actual tonsil volumes [7]. On sonography, the normal palatine tonsils appear hypoechoic to the adjacent submandibular glands. Additionally, the tonsillar crypts give the palatine tonsils their characteristic striated appearance with alternating linear bright and echo poor alternating bands [8]. This normal characteristic appearance adds significant diagnostic value by providing a reliable means to distinguish benign tonsillar hypertrophy from abscess formation and potentially neoplastic or asymmetric enlargement, improving patient care and optimizing the use of more invasive or costly investigations [6].

The lack of ionization radiation and sedation and ease of accessibility with bedside-use feasibility makes US a safe and practical diagnostic tool for tonsillar disease [9]. There are two methods of US evaluation of palatine tonsils, which are transcutaneous and intraoral US and they showed high diagnostic accuracy, without a significant difference between them [9]. Tonsil diseases, such as tonsillitis, peritonsillar abscess, and obstructive sleep apnea are commonly encountered in pediatric clinical practice [10]. Defining normal dimensions and volumes of tonsils by US is crucial for a prompt and precise diagnosis of peritonsillar infections [6, 11].

The tonsils grow during infancy and early childhood due to immune stimulation, peak in size around school-age years, and subsequently undergo natural involution during adolescence, which should be considered when evaluating tonsil size variability. In pediatric clinical practice, tonsillitis, peritonsillar abscess, and obstructive sleep apnea are frequently seen [9]. The timely and accurate diagnosis of peritonsillar infections depends on the US defining normal tonsil sizes and volumes [8, 11]. To the best of our knowledge, no previous research had been conducted in Ethiopia to determine the normal palatine tonsil size in the under 18-year-old population using an US-based measurement. Our aim was thus to estimate the normal palatine tonsil size in under 18-year-old patients who visited TASH for nontonsil-related complaints. Our results would contribute to forming a normative palatine tonsil size for the local under 18-year-old population.

## 2. Materials and Methods

### 2.1. Study Design and Period

A prospective cross-sectional study was employed to determine the sonographic normal palatine size and volume in under 18-year-old patients who visited TASH from May 16–September 30, 2023. TASH is the largest referral hospital in Ethiopia.

### 2.2. Study Population

All under 18-year-old patients who visited the US unit in the study period were included.

### 2.3. Sampling Technique

Total population sampling technique was used.

### 2.4. Inclusion and Exclusion Criteria

All patients who are under the age of 18 years who visited TASH for complaints unrelated to tonsils or do not significantly affect tonsillar size were included in the study. But patients who had tonsillar diseases, tonsillectomy, neck infection, malignancies (such as leukemia and lymphoma), clinical suspicion of airway disease, critical illness, patients who were difficult to scan due to airway disease, or other critical illnesses were excluded. Similarly, parents or patients who refused to give consent were not included in this study.

### 2.5. Study Variables

Palatine tonsil size was the dependent variable. Age, sex, weight, height, body mass index (BMI), and laterality were the independent variables analyzed for association.

### 2.6. Data Collection

A structured questionnaire that contained social, anthropometric, and sonographic measurement parameters for right and left tonsils was uniformly used for all study participants. Palatine tonsil size was determined using Mindray DC 60 US linear high-frequency probe. Weight and height were measured on the same day using portable stadiometer and a digital weighing scale.

The US measurements were performed by the primary investigator Raja Tamiru and three other co-authors of this research (Michael T/Haimanot, Nathnael Alemu, and Henok Dessalegn). All of them were senior radiology residents who had already completed multiple rotations in head and neck ultrasonography. Additionally, a 1-week training session was conducted by a consultant pediatric radiologist to instruct on the measurement procedures. This level of experience ensured that the operators possessed relevant expertise and familiarity with the anatomical structures involved, contributing to methodological reliability. Although interobserver variability was not quantitatively assessed in this study, the use of trained and experienced operators following standardized protocols was intended to minimize measurement variability and enhance consistency across examiners.

**Figure 1:**
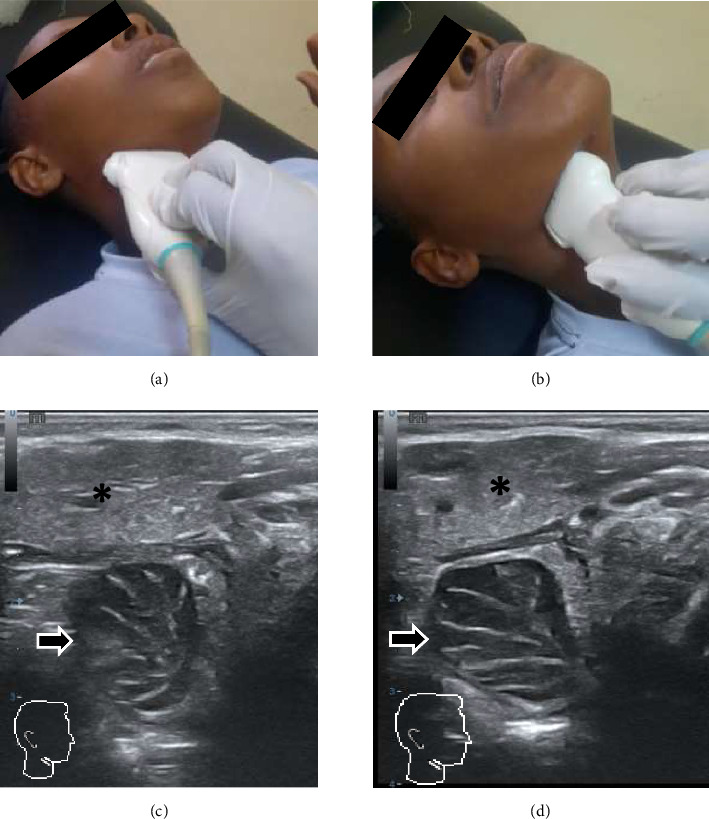
Measurement technique and transcutaneous submandibular sonographic views. Tonsil US performed via submandibular approach on a 10-year-old study participant. The correct placement of the linear transducer in the transverse plane with the probe marker facing the patient's right ear (a) and in the longitudinal plane underneath the mandible (b) are shown. Gray-scale image in transverse (c) and longitudinal planes (d) show the right palatine tonsil (arrows in (c) and (d)) as an ovoid hypoechoic structure located deep to submandibular gland (asterisk in (c) and (d)).

During an examination, patients were put in supine position with the neck slightly extended and the examiner on the side of the patient. For infants, the mother or caretaker holds on their lap with their neck extended. After adequate gel application, a high-frequency linear transducer was used for a transcutaneous submandibular approach. The palatine tonsils were evaluated in both long- and short-axis views; then, measurements were taken in three dimensions and volume was obtained using the abc x 0.52 formula of ellipsoid volume calculation (Figure 1).

### 2.7. Data Analysis and Processing

The collected data were checked for completeness and exported to the statistical package for social science (SPSS) v. 27 for further analysis. Descriptive results are presented using the mean and standard deviation and are presented using table and chart formats. The paired sample *t*-test was used to assess for significant difference in measured means. Additionally, multiple linear regression analysis was employed to examine the influence of anthropometric parameters on palatine tonsil size across different age groups.

## 3. Results

A total of 265 patients under 18 years old were included in the study who visited TASH for tonsil unrelated complaints. Of them, 130 (49%) were male and 135 (51%) were female. The population median age was 6 years with an age range of 0–18 years.

During data analysis, potential outliers in tonsil size within specific age subgroups were carefully examined. No extreme outlier values that could significantly skew the results were identified. Therefore, all measurements were included in the final analysis without exclusion or adjustment for outliers.

The normal palatine tonsil appearance on US was hypoechoic soft tissue with internal linear hyperechoic lines. The palatine tonsils appear hypoechoic as compared with the adjacent submandibular gland.

The mean size of the right palatine tonsil for the whole population was 0.97 cm^3^ ± 0.55 and it was 0.97 cm^3^ ± 0.56 for the left tonsil. The PT measurement normal values (mean ± standard deviation) based on age groups are presented on Tables 1 and 2.

The measurement of PT diameters and PT volume were similar for boys and girls as suggested by an independent samples *t*-test (*p*=0.61). Similarly, the paired samples *T*-test showed that the measurements have insignificant difference for right and left side PT (*p*=0.71). Based on correlation analysis, significant correlation was detected between PT values and age, weight, height, and BMI. Height has the strongest correlation while BMI has the least correlation with PT values (Table 3).

**Figure 2:**
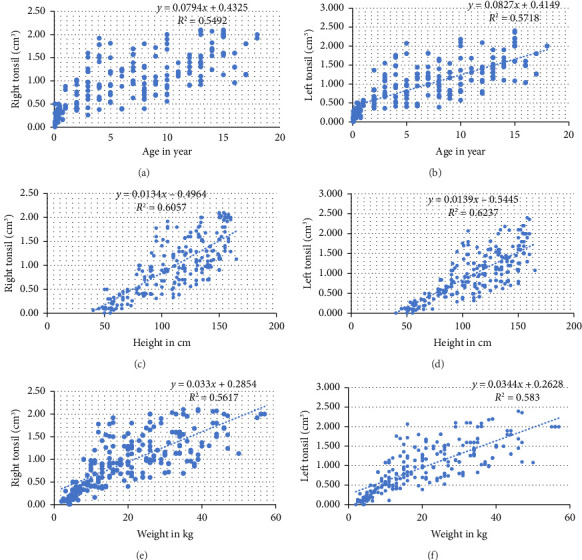
Scatter plot of anthropometric measurements relative to the size of right/left palatine tonsil based on single linear regression analysis. (a-b) Scatter plots of age and size of the right/left palatine tonsil. (c-d) A scatter plot of the height and size of the right/left palatine tonsil, and (e-f) a scatter plot of the weight and size of the right/left palatine tonsil.

A scatter plot of the right/left tonsil size in relation to age, weight, height, and BMI can be seen in Figure 2.

We examined the influence of anthropometric parameters on palatine tonsil size across different age groups using multiple linear regression analysis. The standardized regression coefficients (Beta) and corresponding *p* values are summarized in Table 4.

For children under 1 year, height was the strongest and a statistically significant predictor of tonsil size (*β* = 0.571, *p*=0.002). In the 1–4-year age group, weight showed the strongest and highly significant association (*β* = 0.8, *p* < 0.001). Among children aged 5–9 years, height again had the highest significant influence on tonsil size (*β* = 1.4, *p*=0.03). For ages 10–14 years, weight presented the largest *β* (0.9), but the association did not reach statistical significance (*p*=0.12), indicating only a suggestive relationship. In participants aged 15 years and older, BMI showed the highest *β* (3.2) but was not statistically significant (*p*=0.3), precluding definitive conclusions regarding its effect on tonsil size.

These results indicate that the relative contribution of anthropometric parameters to tonsil size varies with age: height is more influential in younger children, weight in early childhood, whereas evidence for BMI influence in older individuals is inconclusive.

## 4. Discussion

Ultrasonography has been used for evaluation of tonsillar infections. It is simple noninvasive technique that can precisely differentiate peritonsillar abscesses from peritonsillar cellulitis. Moreover, it also avoids blind needle aspiration of peritonsillar abscess and its associated potential complications [7, 8, 12].

A study done by Miziara et al. showed 92.3% sensitivity and 62.5% specificity of intraoral US in the diagnosis of peritonsillar abscess [13]. On the other hand, Filho and et al. reported comparable accuracy of transcutaneous and intraoral US [14].

CT and MRI can be used for the evaluation of tonsillar pathologies. CT is indicated for evaluation of tonsilitis and abscess or extratonsillar infection spread assessment. The main drawback of using CT is its associated ionized radiation that highly affects radiosensitive organs in the scanning field such as the thyroid gland [15]. MRI provides a better soft tissue resolution and it does not contain ionizing radiation. However, the requirement for sedation, cost, and relative unavailability are downsides of MRI. Use of US decreases the exposure to ionizing radiation, is less expensive, and easily available as compared with CT or MRI. Defining normal dimensions and volumes of tonsils by US is crucial for a prompt and precise diagnosis of peritonsillar infections [6, 11]. However, little information is available regarding the normal values of PT size and volume.

This study defines normal PT measurements and volume in the ages between 0 and 18 years using ultrasonography. This study provides estimated PT measurements that can be used for the diagnosis of tonsillar infections and other pathologies associated with PT size alteration. According to our results, the mean PT volume is 0.97 cm^3^ ± 0.55 for the right tonsil and 0.97 cm^3^ ± 0.56 for the left tonsil. A study on exactly same age groups by Hong et al. showed comparable mean PT volume of 0.85 ± 0.75 cm^3^ for the right tonsil and 0.75 ± 0.56 cm^3^ for the left tonsil [9]. These results are smaller as compared with Sonay Aydin's and Hsokawa et al.'s studies [6, 10]. Palatine tonsil size measurements can show variability across different studies due to several influential factors. One significant factor is ethnicity, as anatomical differences in lymphoid tissues, including tonsils, may exist among populations with different genetic backgrounds and environmental influences. Additionally, anthropometric distributions vary among populations, which also contribute to palatine tonsil size variability. Such variations can affect baseline tonsil size, contributing to differences observed in studies conducted in diverse ethnic groups. Another key factor relates to differences in US protocols and techniques. Variation in transducer frequency, patient positioning, probe handling, and measurement approaches can influence tonsil size estimations. Operator experience and interpretation criteria also play a role in measurement consistency, explaining part of the variability seen across studies. Taken together, these factors emphasize the need for standardized measurement protocols, clear reporting on population characteristics, and careful consideration of demographic differences when interpreting and comparing palatine tonsil measurements across studies [11, 16].

We found that for children under the age of 5, tonsil sizes increase with age. Our findings agreed with the study by Sonay Aydin as well as Jaw et al., which found that tonsillar diameters had plateau in the age range of 2–7 years [6, 17].

According to our results, a significant positive correlation was found between the PT size and height, weight, and BMI. These findings were also evidenced by other prior studies by Hosowaka et al., Sonay Aydin et al., and Zohida Abdel et al. [6, 10, 18]. In our study, height has the strongest positive correlation with PT size while BMI had the weakest correlation. This finding is consistent with the findings of Hosokawa et al. [10]. However, Hong et al. reported opposing result in which they found no correlation for height and weight. This difference in the result can be due to smaller number of participants as compared with ours (161 vs. 265, respectively).

Our study highlights the varying influence of anthropometric parameters on palatine tonsil size through different childhood and adolescent age groups. For infants under 1 year, height emerged as the strongest anthropometric predictor of tonsil size, consistent with previous findings that height correlates significantly with tonsillar development in early childhood. This may reflect general body growth influencing lymphoid tissue size in infancy [19].

In the 1–4-year age group, weight was the dominant factor showing a significant association with tonsil size. This is in line with reports that the weight status plays a larger role in younger children's tonsillar hypertrophy and related obstructive airway issues. Increased body weight and adiposity in this period may contribute to systemic inflammatory processes that affect the tonsil size and function [20, 21].

For children aged 5–9 years, height again showed a significant positive influence on tonsil size. This supports work demonstrating that during the midchildhood growth phase, linear growth remains a key determinant of tonsil volume and potentially adenotonsillar hypertrophy. This phase coincides with increased lymphoid tissue proliferation, which is a natural part of immune system maturation [22, 23].

In preadolescents aged 10–14 years, weight had the highest standardized effect size on tonsil size but did not reach statistical significance. This suggests a potential trend that warrants further larger studies to clarify weight's role during the onset of puberty when growth and hormonal changes may impact tonsillar tissue differently [21, 24].

Finally, for adolescents aged 15 years and above, BMI showed the largest but nonsignificant association with tonsil size. While BMI is known to be associated with tonsillar hypertrophy and related conditions such as obstructive sleep apnea in adults, our findings align with studies reporting inconsistent or weak correlations of BMI with tonsil size in older age groups. These inconsistencies may be due to varying adiposity distributions or other systemic factors that become more relevant postadolescence [19].

In line with previous research studies, we were unable to identify any differences in PT size between sexes or between right and left sizes [9, 10, 25]. Therefore, any variation in tonsil diameters between two sides may serve as pathology warning signal. Furthermore, during tonsillar US, it is unnecessary to take into account sex of patients for difference in PT size.

Intra- and inter-rater variabilities are important considerations in US measurements due to their impact on the reliability and reproducibility of the results. In US assessment of the palatine tonsil, variability can arise from differences in probe positioning, applied pressure, and interpretation of anatomical landmarks both within a single operator's repeated measurements (intrarater variability) and between different operators (inter-rater variability). The anatomical complexity and small size of the palatine tonsil may further contribute to measurement inconsistency. Although this study did not quantify variability statistically, rigorous protocols were employed to minimize potential inconsistencies, including standardized scanning techniques and training of operators. A previous study by Abdelgabar et al. has reported moderate to excellent reliability in similar US measurements, highlighting that while variability is inherent, it can be effectively managed through methodical approaches [11]. Nonetheless, future studies are encouraged to include formal quantification of intra- and inter-rater reliability to further validate the robustness of US as a tool for palatine tonsil assessment.

## 5. Conclusion

Tonsillar US is a well-tolerated, noninvasive procedure that can be used in pediatric populations. Its use not only avoids radiation exposure but also helps radiologists identify tonsillar pathologies accurately. Tonsillar lesions can be diagnosed using these normal values. In this study, we provided estimated measurement values for normal right and left palatine tonsils of patients in the age range of 0–18 years. Over the first 5 years, we observed a considerable increase in tonsil size. There was a relationship between tonsil size and anthropometric indices such as age, weight, and height and BMI with height exhibiting the most robust association. This study also demonstrates that the influence of anthropometric parameters on palatine tonsil size varies with age. Height is the strongest predictor in infants and midchildhood, while weight is more influential during early childhood. In older children and adolescents, the associations with weight and BMI are less consistent or statistically nonsignificant, suggesting more complex developmental factors at play. These findings highlight the importance of considering age-specific anthropometric profiles when assessing tonsil size and related clinical conditions. Additionally, ethnic population differences also contribute to variability in palatine tonsil size measurements and should be carefully considered when interpreting normal tonsil dimensions.

## 6. Limitations of the Study

There are few noteworthy drawbacks to this study. The use of a single study area and the relatively new practice of using US for tonsillar assessment in our institution are the limitations of the study. This research seeks to define normal tonsil size in patients with no tonsillar pathologies. Studying alongside both normal and with tonsillar diseases can facilitate to define a threshold value to distinguish between normal as well as pathologic tonsil sizes. Concurrent clinical oral examination was not done alongside the US measurements of palatine tonsils which would help to assess correlation between the clinical and sonographic measurements. Given that tonsil size and anthropometric parameters are correlated, normal values may alter with various societies and/or nations.

## 7. Recommendation

This study aims to estimate tonsil size in healthy children only. Studying with both healthy children and the ones with tonsillar pathologies can enable researchers to define cutoff values to differentiate normal and abnormal tonsil sizes. Since this study was done in Ethiopia, future studies are recommended including populations from other countries or ethnic groups to account for variability of palatine tonsil size differences among different races. Future research should also include formal quantification of intra- and inter-rater variability to further validate and strengthen the reliability of US measurements of the palatine tonsil. This will enhance reproducibility and support wider clinical application of this imaging technique.

## Figures and Tables

**Table 1:** Measured right palatine tonsil size.

Age (years)	Patient number	Palatine tonsil diameters (cm)			Palatine tonsil volume (cm^3^)
		AP	Transverse	Longitudinal	
< 1	47	0.60 ± 0.014	0.67 ± 0.16	0.79 ± 0.15	0.21 ± 0.14
1–4	60	1.01 ± 0.19	1.12 ± 0.19	1.36 ± 0.30	0.86 ± 0.42
5–9	78	1.10 ± 0.16	1.20 ± 0.017	1.43 ± 0.25	1.02 ± 0.34
10–14	50	1.16 ± 0.15	1.32 ± 0.21	1.61 ± 0.24	1.34 ± 0.40
≥ 15	30	1.28 ± 0.09	1.41 ± 0.14	1.69 ± 0.29	1.64 ± 0.38

**Table 2:** Measured left palatine tonsil size.

Age (years)	Patient number	Mean palatine tonsil diameters (cm)			Mean palatine tonsil volume (cm^3^)
		AP	Transverse	Longitudinal	
< 1	47	0.60 ± 0.13	0.67 ± 0.14	0.81 ± 0.17	0.19 ± 0.13
1–4	60	1.04 ± 0.18	1.13 ± 0.20	1.32 ± 0.26	0.87 ± 0.40
5–9	78	1.09 ± 0.18	1.20 ± 0.20	1.42 ± 0.22	1.01 ± 0.35
10–14	50	1.25 ± 0.28	1.31 ± 0.18	1.57 ± 0.23	1.33 ± 0.38
≥ 15	30	1.28 ± 0.11	1.50 ± 0.13	1.68 ± 0.29	1.73 ± 0.39

**Table 3:** Correlations between palatine tonsil size and anthropometric parameters.

		Age	Weight	Height	BMI
Right tonsil volume	*r*	0.74	0.75	0.78	0.30
	*p*	< 0.001	< 0.001	< 0.001	< 0.001

Left tonsil volume	*r*	0.76	0.76	0.80	0.30
	*p*	< 0.001	< 0.001	< 0.001	< 0.001

**Table 4:** Anthropometric predictors of palatine tonsil size by age group.

Age (years)	Parameter with highest standardized beta	Standardized beta (*β*)	*p* value	Statistical significance
< 1	Height	0.571	0.002	Significant
1–4	Weight	0.8	< 0.001	Significant
5–9	Height	1.4	0.03	Significant
10–14	Weight	0.9	0.12	Not significant (suggestive)
≥ 15	BMI	3.2	0.3	Not significant

## Data Availability

The data of measurements are available, which will be presented upon request to the corresponding author.

## Nomenclature

AAU: Addis Ababa University
BMI: Body mass index
CT: Computed tomography
MRI: Magnetic resonance imaging
TASH: Tikur Anbessa Specialized Hospital
US: Ultrasound
